# Supplementary material for: Patronin governs minus-end-out orientation of dendritic microtubules to promote dendrite pruning in Drosophila
Source: eLife. 2019 Mar 28;8:e39964. doi: 10.7554/eLife.39964 (PMC6438692; doi:10.7554/eLife.39964)
Supplement: Supplementary file 1. [file elife-39964-supp1.docx]

**Supplemental list of fly strains**

**Figure 1:** (B) *w*;; ppk-Gal4, UAS-mCD8GFP / ppk-Gal4, UAS-mCD8GFP.* (C) *w*; ppk-Gal4, UAS-mCD8GFP* / *UAS-patr* RNAi #1*; UAS-Dcr2* / *+*. (D) *w*; FRT G13, patr^c9-c5^* / *FRT G13, tubP-Gal80; ppk-Gal4, UAS-mCD8­GFP, SOP-flp* / *+.* (E) *w*; FRT G13, patr^c9-c5^* / *FRT G13, tubP-Gal80; ppk-Gal4, UAS-mCD8­GFP, SOP-flp* / *UASp-mCherry-Patr.* (F) *w*; FRT 42D, patr^k07433^* / *FRT 42D, tubP-Gal80; ppk-Gal4, UAS-mCD8­GFP, SOP-flp* / *+.*

**Figure 2:** (A) *w*; ppk-Gal4* / *+ ; ppk-Gal4, UAS-mCD8GFP* / *UAS-Mical^NT^.* (B) *w*; Gal4^4-77^, UAS-mCD8GFP* / *+ ; UAS-Patr* / *+.* (C) *w*; ppk-Gal4* / *+ ; ppk-Gal4, UAS-mCD8GFP* / *UAS-GFP-Patr.* (D): *w*;* GSG2295-*Gal4, ppk-CD4-tdGFP / +;* *UAS-GFP-Patr / +****.*** (E): *w*;* GSG2295-*Gal4, ppk-CD4-tdGFP* */ +;* *UAS-GFP-Patr / +****.***

**Figure 3:** (A) *w*; FRT G13, patr^c9-c5^* / *FRT G13, tubP-Gal80; ppk-Gal4, UAS-mCD8­GFP, SOP-flp* / *+.* (B) *w*; FRT G13, patr^c9-c5^* / *FRT G13, tubP-Gal80; ppk-Gal4, UAS-mCD8­GFP, SOP-flp* / *UASp-Patr^ΔCH^.* (C) *w*; FRT G13, patr^c9-c5^* / *FRT G13, tubP-Gal80; ppk-Gal4, UAS-mCD8­GFP, SOP-flp* / *UASp-Patr^ΔCKK^.* (D) *w*; FRT G13, patr^c9-c5^* / *FRT G13, tubP-Gal80; ppk-Gal4, UAS-mCD8­GFP, SOP-flp* / *UASp-Patr^CKK^.* (G) *w*; Gal4^4-77^, UAS-mCD8GFP* / *+ ; UAS-Venus-Patr* / *+.* (H) *w*; Gal4^4-77^, UAS-mCD8GFP* / *+ ; UAS-Venus-Patr^ΔCH^* / *+.* (I) *w*; Gal4^4-77^, UAS-mCD8GFP* / *+ ; UAS-Venus-Patr^ΔCKK^*/ *+.* (J) *w*; Gal4^4-77^, UAS-mCD8GFP* / *+ ; UAS-Venus-Patr^CKK^* / *+.*

**Figure 4:** (A) *w*; ppk-Gal4, UAS-mCD8GFP* / *+ ; UAS-Nod-β-gal*  / *UAS-Mical^NT^.* (B) *w*; ppk-Gal4, UAS-mCD8GFP, UAS-Dcr2* / *UAS-patr* RNAi #1*; UAS-Nod-β-gal* / +. (C) *w*; ppk-Gal4, UAS-mCD8GFP* / *+; UAS-Nod-β-gal*/*UAS-GFP-Patr.* (F) *w*; ppk-Gal4, UAS-mCD8GFP, UAS-Dcr2* / *+ ; UAS-Kin-β-gal*  / *UAS-control* RNAi (v37288)*.* (G) *w*; ppk-Gal4, UAS-mCD8GFP, UAS-Dcr2* / *UAS-patr* RNAi #1*; UAS-Kin-β-gal* / *+.* (H) *w*; ppk-Gal4, UAS-mCD8GFP* / *+ ; UAS-Kin-β-gal*  / *UAS-GFP-Patr.*

**Figure 5:** (A) *w*; Gal4^4-77^, UAS-EB1-GFP* / *+; UAS-Dcr2* / *UAS-*control RNAi (v37288)*.* (B) *w*; Gal4^4-77^, UAS-EB1-GFP* / *UAS-patr* RNAi #1*; UAS-Dcr2* / +. (G) *w*; Gal4^4-77^, UAS-EB1-GFP* / *+ ; UAS-Mical^NT^* / *+.* (H) *w*; Gal4^4-77^, UAS-EB1-GFP* / *+; UAS-Patr* / *+.*

**Figure 6:** (A) *w*; Gal4^4-77^, UAS-EB1-GFP* / *UAS-patr* RNAi #1*; UAS-Dcr2* / *UAS-*control RNAi (v37288)*.* (B) *w*; Gal4^4-77^, UAS-EB1-GFP* / *UAS-patr* RNAi #1*; UAS-Dcr2* / *UAS-klp10A* RNAi #1*.* (G) *w*; ppk-Gal4, UAS-mCD8GFP* / *+ ; UAS-Nod-β-gal*  / *UAS-Mical^NT^.* (H) *w*; ppk-Gal4, UAS-mCD8GFP, UAS-Dcr2* / *UAS-patr* RNAi #1*; UAS-Nod-β-gal* / *UAS-*control RNAi (v37288)*.* (I) *w*; ppk-Gal4, UAS-mCD8GFP, UAS-Dcr2* / *UAS-patr* RNAi #1*; UAS-Nod-β-gal* / *UAS-klp10A* RNAi #1*.* (L) *w*; ppk-Gal4, UAS-mCD8GFP, UAS-Dcr2* / *+; UAS-Kin-β-gal* / *UAS-*control RNAi (v37288)*.* (M) *w*; ppk-Gal4, UAS-mCD8GFP, UAS-Dcr2* / *UAS-patr* RNAi #1*; UAS- Kin-β-gal*  / *UAS-*control RNAi (v37288)*.* (N) *w*; ppk-Gal4, UAS-mCD8GFP, UAS-Dcr2* / *UAS-patr*  RNAi #1 *; UAS- Kin-β-gal*  / *UAS-klp10A* RNAi #1*.*

**Figure 7:** (A) *w*; ppk-Gal4, UAS-mCD8GFP, UAS-Dcr2* / *UAS-patr* RNAi #1*; UAS-control* RNAi (v36355) / *+.* (B) *w*; ppk-Gal4, UAS-mCD8GFP, UAS-Dcr2* / *UAS-patr* RNAi #1 *; UAS-klp10A* RNAi #1 / *+.* (C) *w*; ppk-Gal4, UAS-mCD8GFP, UAS-Dcr2* / *UAS-patr* RNAi #1 *; UAS-klp10A* RNAi #2 / *+.* (F) *w*; ppk-Gal4, UAS-mCD8GFP, UAS-Dcr2* /+ *; UAS-control* RNAi (v36355) / *UAS-patr.* (G) *w*; ppk-Gal4, UAS-mCD8GFP, UAS-Dcr2* /+ *; UAS-klp10A* RNAi #1 / *UAS-patr.* (H) *w*; ppk-Gal4, UAS-mCD8GFP, UAS-Dcr2* /+ *; UAS-klp10A* RNAi #2 / *UAS-patr.* (K) *w*; Gal4^4-77^, UAS-EB1-GFP* / *+; UAS-control* RNAi (v36355) / *UAS-patr.* (L) *w*; Gal4^4-77^, UAS-EB1-GFP* / *+; UAS-klp10A* RNAi #2 / *UAS-patr.*

**Figure 8:** (A) *w*; ppk-Gal4* / *+; ppk-Gal4, UAS-mCD8GFP* / *UAS-Mical^NT^.* (B) *w*; ppk-Gal4* / *+; ppk-Gal4, UAS-mCD8GFP* / *UAS-GFP-Klp10A.* (C) *w*; ppk-Gal4* / *+; ppk-Gal4, UAS-mCD8GFP* / *UAS-Klp10A.* (F) *w*; Gal4^4-77^, UAS-EB1-GFP* / *+; UAS-Mical^NT^* / *+.* (G) *w*; Gal4^4-77^, UAS-EB1-GFP* / *+; UAS-Klp10A* / *+.*

**Figure 1–figure supplement 1:** (A) **Control:** *w*; ppk-Gal4, UAS-mCD8GFP, UAS-Dcr2* / *ppk-Gal4, UAS-mCD8GFP, UAS-Dcr2; UAS-control* RNAi (v36355) / *UAS-control* RNAi (v36355)*.* ***patr* RNAi #2:** *w*; ppk-Gal4, UAS-mCD8GFP, UAS-Dcr2*/ *ppk-Gal4, UAS-mCD8GFP, UAS-Dcr2; UAS-patr* RNAi #2 / *UAS-patr* RNAi #2*.* ***patr* RNAi #3:** *w*; ppk-Gal4, UAS-mCD8GFP, UAS-Dcr2*/ *ppk-Gal4, UAS-mCD8GFP, UAS-Dcr2; UAS-patr* RNAi #3 / *UAS-patr* RNAi #3*.* (B) **Control:** *w*; ppk-Gal4, UAS-mCD8GFP, UAS-Dcr2* /*+ ; UAS-control* RNAi (v36355) / *+.* ***patr* RNAi #1:** *w*; ppk-Gal4, UAS-mCD8GFP, UAS-Dcr2* / *UAS-patr* RNAi #1*.* (C) **FRT G13 Control:** *w*; FRT G13* / *FRT G13, tubP-Gal80; ppk-Gal4, UAS-mCD8­GFP, SOP-flp* / *+.* ***patr^c9-c5^*:** *w*; FRT G13, patr^c9-c5^* / *FRT G13, tubP-Gal80; ppk-Gal4, UAS-mCD8­GFP, SOP-flp* / *+.*  (D) **Control (ddaD/E):** *w*;; Gal4^2-21^ ,UAS-mCD8GFP / Gal4^2-21^ ,UAS-mCD8GFP.* ***patr* RNAi** **(ddaD/E):** *w*; UAS-patr* RNAi #1 */ UAS-Dcr2; Gal4^2-21^, UAS-mCD8GFP /+.* (E) **Control (ddaF):** *w*; Gal4^109(2)80^, UAS-mCD8GFP/ Gal4^109(2)80^, UAS-mCD8GFP; UAS-Dcr2* / *UAS- Dcr2.* ***patr* RNAi** #1 **(ddaF):**  *w*; Gal4^109(2)80^, UAS-mCD8GFP* / *UAS-patr*  RNAi #1*; UAS-Dcr2* /*+*.

**Figure 2-figure supplement 1:** (A) **Control:** *w*; ppk-Gal4* / *+; ppk-Gal4, UAS-mCD8GFP* / *UAS-Mical^NT^.* **O/E *UASp-mCherry-Patr*:** *w*; ppk-Gal4* / *+; ppk-Gal4, UAS-mCD8GFP* / *UASp-mCherry-Patr.* **O/E *UAS-GFP-Patr*:** *w*; ppk-Gal4* / *+ ; ppk-Gal4, UAS-mCD8GFP* / *UAS-GFP-Patr.* **O/E *UAS-Patr*:** *w*; ppk-Gal4* / *+ ; ppk-Gal4, UAS-mCD8GFP* / *UAS-Patr.* (B) **O/E *UAS* Control:** *w*; ppk-Gal4* / *+; ppk-Gal4, UAS-mCD8GFP* / *UAS-Mical^NT^.* **O/E *UAS-GFP-Patr***: *w*; ppk-Gal4* / *+; ppk-Gal4, UAS-mCD8GFP* / *UAS-GFP-Patr.*

**Figure 3-figure supplement 1:** (B) **O/E UASp Control:** *w*; ppk-Gal4* / *+ ; ppk-Gal4, UAS-mCD8GFP* / *UASp-Arf79F-EGFP.* **O/E *UASp-mCherry-Patr:*** *w*; ppk-Gal4* / *+ ; ppk-Gal4, UAS-mCD8GFP* / *UASp-mcherry-Patr.* **O/E *UASp-Patr^ΔCH^*:** *w*; ppk-Gal4* / *+ ; ppk-Gal4, UAS-mCD8GFP* / *UASp-Patr^ΔCH^*. **O/E *UASp-Patr^ΔCKK^*:** *w*; ppk-Gal4* / *+ ; ppk-Gal4, UAS-mCD8GFP* / *UASp-Patr^ΔCKK^*. **O/E *UASp-Patr^CKK^*:** *w*; ppk-Gal4* / *+ ; ppk-Gal4, UAS-mCD8GFP* / *UASp-Patr^CKK^*.

**Figure 4-figure supplement 1:** (A) **FRT Control:** *w*; ppk-Gal4, UAS-mCD8­GFP, SOP-flp* / +*; FRT82B, UAS-Nod-β-gal* / *FRT82B, tubP-Gal80.* ***patr^c9-c5^***: *w*; FRT G13, patr^c9-c5^* / *FRT G13, tubP-Gal80; ppk-Gal4, UAS-mCD8­GFP, SOP-flp* / *UAS-Nod-β-gal .* (B) **96 h AEL:** *w*; ppk-Gal4* / *+ ; ppk-Gal4, UAS-mCD8GFP*/ *+.* **WP:** *w*; ppk-Gal4* / *+ ; ppk-Gal4, UAS-mCD8GFP*/ *+.*

**Figure 5-figure supplement 1:** (A) **Control RNAi:** *w*; Gal4^4-77^, UAS-EB1-GFP* / *+; UAS-Dcr2* / *UAS-*control RNAi (v37288)*.* ***patr* RNAi #2:** *w*; Gal4^4-77^, UAS-EB1-GFP* / *+ ; UAS-patr* RNAi #2 / *+.* (B) **Control RNAi:** *w*; ppk-Gal4, UAS-mCD8GFP, UAS-Dcr2* / *+ ; UAS-control* RNAi (v36355) / *+.* ***patr* RNAi #1:** *w*; ppk-Gal4, UAS-mCD8GFP, UAS-Dcr2* / *UAS-patr* RNAi #1*.* (C) **Control RNAi:** *w*; Gal4^4-77^, UAS-EB1-GFP* / *+ ; UAS-Dcr2* / *UAS-*control RNAi (v37288)*.* ***patr* RNAi #1:** *w*; Gal4^4-77^, UAS-EB1-GFP* / *UAS-patr*  RNAi #1 *; UAS-Dcr2* / +.

**Figure 6-figure supplement 1:** (B) **Control RNAi:** *w*; Gal4^4-77^, UAS-EB1-GFP* / *+; UAS-Dcr2* / *UAS-*control RNAi (v37288)*.* ***klp10A* RNAi #1:** *w*; Gal4^4-77^, UAS-EB1-GFP* / *+; UAS-Dcr2* / *UAS-klp10A* RNAi #1*.* (C) **Control:** *w*; ppk-Gal4, UAS-mCD8GFP, UAS-Dcr2* / *+; UAS-Nod-β-gal* / *UAS-*control RNAi (v36355)*.* ***klp10A* RNAi #1:** *w*; ppk-Gal4, UAS-mCD8GFP, UAS-Dcr2* / *+; UAS-klp10A* RNAi #1 / *UAS-Nod-β-gal.* ***klp10A* RNAi #2:** *w*; ppk-Gal4, UAS-mCD8GFP, UAS-Dcr2* / *+ ; UAS-klp10A* RNAi #2 / *UAS-Nod-β-gal.*

**Figure 7-figure supplement 1:** (A) **Control:** *w*; ppk-Gal4, UAS-mCD8GFP, UAS-Dcr2* / *+; UAS-control* RNAi (v36355) / *+.* ***klp10A* RNAi #1:** *w*; ppk-Gal4, UAS-mCD8GFP, UAS-Dcr2* / *+; UAS-klp10A* RNAi #1/ *+.* ***klp10A* RNAi #2:** *w*; ppk-Gal4, UAS-mCD8GFP, UAS-Dcr2* / *+; UAS-klp10A* RNAi #2 /*+.* (B) **Control:** *w*; ppk-Gal4, UAS-mCD8GFP, UAS-Dcr2* / *+; UAS-control* RNAi (v36355) / *+.* ***Kat60* RNAi #1:** *w*; ppk-Gal4, UAS-mCD8GFP, UAS-Dcr2* / *+; UAS-Kat60* RNAi #1/ *+.* ***Kat60* RNAi #2:** *w*; ppk-Gal4, UAS-mCD8GFP, UAS-Dcr2* / *UAS-Kat60* RNAi #2; + / +*.* (C) ***patr* RNAi #1 *+* Control RNAi:** *w*; ppk-Gal4, UAS-patr* RNAi #1 / *+;* *ppk-Gal4, UAS-mCD8GFP, UAS-Dcr2 / UAS-control* RNAi (v36355). ***patr* RNAi #1 *+ kat60* RNAi #1:** *w*; ppk-Gal4, UAS-patr* RNAi #1 / *+;* *ppk-Gal4, UAS-mCD8GFP, UAS-Dcr2 / UAS-kat60* RNAi #1. ***patr* RNAi #1 *+ kat60* RNAi #2:** *w*; ppk-Gal4, UAS-patr* RNAi #1 / *UAS-kat60* RNAi #2*;* *ppk-Gal4, UAS-mCD8GFP, UAS-Dcr2 / +*. (D) ***patr* RNAi #1 *+* Control RNAi:** *w*; ppk-Gal4, UAS-patr* RNAi #1 / *+;* *ppk-Gal4, UAS-mCD8GFP, UAS-Dcr2 / UAS-control* RNAi (v36355). ***patr* RNAi #1 *+ kat60L1* RNAi #1:** *w*; ppk-Gal4, UAS-patr* RNAi #1 / *UAS-kat60L1* RNAi #1*;* *ppk-Gal4, UAS-mCD8GFP, UAS-Dcr2 / +*. ***patr* RNAi #1 *+ kat60* *L1* RNAi #2:** *w*; ppk-Gal4, UAS-patr* RNAi #1 / *UAS-kat60L1* RNAi #2*;* *ppk-Gal4, UAS-mCD8GFP, UAS-Dcr2 /+*. (E) ***patr* RNAi #1 *+* Control RNAi:** *w*; ppk-Gal4, UAS-patr* RNAi #1 / *+;* *ppk-Gal4, UAS-mCD8GFP, UAS-Dcr2 / UAS-control* RNAi (v36355). ***patr* RNAi #1 *+ tau* RNAi #1:** *w*; ppk-Gal4, UAS-patr* RNAi #1 /*+;* *ppk-Gal4, UAS-mCD8GFP, UAS-Dcr2 / tau*  RNAi #1. ***patr* RNAi #1 *+ tau* RNAi #2:** *w*; ppk-Gal4, UAS-patr* RNAi #1 / *tau*  RNAi #2*;* *ppk-Gal4, UAS-mCD8GFP, UAS-Dcr2 / +*.

**Figure 8-figure supplement 1:** (A) **Control:** *w*; ppk-Gal4, UAS-mCD8GFP* / *+; UAS-Nod-β-gal*  / *UAS-Mical^NT^.* **O/E *UAS-Klp10A*:** *w*; ppk-Gal4, UAS-mCD8GFP* / *+; UAS-Nod-β-gal*  / *UAS-Klp10A.* (B) **Control:** *w*; ppk-Gal4, UAS-mCD8GFP* / *+ ; UAS-Kin-β-gal*  / *UAS-Mical^NT^.* **O/E *UAS-Klp10A*:** *w*; ppk-Gal4, UAS-mCD8GFP* / *+ ; UAS-Kin-β-gal*  / *UAS-Klp10A.*

**Figure 8--figure supplement 2:** (A) **O/E UASp Control *+ patr* RNAi #1:** *w*; ppk-Gal4, UAS-patr* RNAi #1 / *+;* *ppk-Gal4, UAS-mCD8GFP, UAS-Dcr2 / UASp-Arf79F-EGFP*. **O/E *UASp-Patr-CKK* *+ patr* RNAi #1:** *w*; ppk-Gal4, UAS-patr* RNAi #1 / *+;* *ppk-Gal4, UAS-mCD8GFP, UAS-Dcr2 /UASp-Patr^CKK^*. (B) **O/E UAS Control *+ patr* RNAi #1:** *w*; ppk-Gal4, UAS-mCD8GFP, UAS-Dcr2* / *UAS-patr* RNAi #1*; UAS-Nod-β-gal* / *UAS-Mical^NT^.* **O/E *UASp-Patr-CH + patr* RNAi #1:** *w*; ppk-Gal4, UAS-mCD8GFP, UAS-Dcr2* / *UAS-patr* RNAi #1*; UAS-Nod-β-gal* / *UASp-Patr-CH.* **O/E *UASp-Patr-CKK + patr* RNAi #1:** *w*; ppk-Gal4, UAS-mCD8GFP, UAS-Dcr2* / *UAS-patr* RNAi #1*; UAS-Nod-β-gal* / *UASp-Patr-CKK.* (C) **O/E UAS Control *+ patr* RNAi #1:** *w*; Gal4^4-77^, UAS-EB1-GFP* / *UAS-patr* RNAi #1*; UAS-Dcr2* / *UAS-Mical^NT^.* **O/E *UASp-Patr-CH+ patr* RNAi #1:** *w*; Gal4^4-77^, UAS-EB1-GFP* / *UAS-patr* RNAi #1*; UAS-Dcr2* / *UASp-Patr-CH*. **O/E *UASp-Patr-CKK + patr* RNAi #1:** *w*; Gal4^4-77^, UAS-EB1-GFP* / *UAS-patr* RNAi #1*; UAS-Dcr2* / *UASp-Patr-CKK.* (D) **O/E UASp Control *+*** / **O/E** ***UAS-Klp10A****: w*; ppk-Gal4, UAS-mCD8GFP* / *+; UASp-Arf79F-EGFP* / *UAS-Klp10A.* **O/E *UASp-Patr-CKK +*** **O/E** ***UAS-Klp10A****: w*; ppk-Gal4, UAS-mCD8GFP* / *+ ; UASp-Patr-CKK* / *UAS-Klp10A.*

**Figure 8-figure supplement 3:** (A) **Control:** *w*; +* / *+; ppk-Gal4, UAS-mCD8GFP* /*+.* ***cnn^hk21^*:** *w*; cnn^hk21^* / *cnn^hk21^; ppk-Gal4, UAS-mCD8GFP* /+ (B) **Control:** *w*; ppk-Gal4, UAS-mCD8­GFP, SOP-flp* / +*; FRT82B* / *FRT82B, tubP-Gal80.* ***APC1/2 MARCM* :** *w*; ppk-Gal4, UAS-mCD8­GFP, SOP-flp* / + *; FRT82B, APC2^N175K^, APC2^Q8^* / *FRT82B, tubP-Gal80.* (C) **Control:** *w*; ppk-Gal4, UAS-mCD8GFP, UAS-Dcr2* / *+; UAS-control* RNAi (v36355) / *+.* ***tau* RNAi #1:** *w*; ppk-Gal4, UAS-mCD8GFP, UAS-Dcr2* / *+ ; UAS-tau* RNAi #1 / *+.* ***tau* RNAi #2:** *w*; ppk-Gal4, UAS-mCD8GFP, UAS-Dcr2* / *UAS-tau* RNAi #2*.* (D) **Control:** *w*; ppk-Gal4* / *+ ; ppk-Gal4, UAS-mCD8GFP* /*+.* ***futsch^N94^*:** *w*, futsch^N94^/Y; ppk-Gal4,* /*+; ppk-Gal4, UAS-mCD8GFP* /+.
